# Sociocultural and structural influences on HIV Pre-Exposure Prophylaxis (PrEP) Engagement and Uptake among African American Young adults

**DOI:** 10.1186/s12889-023-16273-8

**Published:** 2023-07-26

**Authors:** Suur D. Ayangeakaa, Jelani Kerr, Ryan M. Combs, Lesley M. Harris, Jeanelle S. Sears, Kimberly Parker, Emma Sterrett-Hong

**Affiliations:** 1 Department of Population Health Sciences, Duke University School of Medicine, 215 Morris St. Durham, Durham, NC 27701 USA; 2 Duke Global Health Institute, Duke University, Durham, NC USA; 3 Department of Health Promotion and Behavioral Sciences, School of Public Health and Information Sciences, University of Louisville, Louisville, KY USA; 4 Kent School of Social Work, University of Louisville, Louisville, KY USA; 5 Department of Human Services, Bowling Green State University, Bowling Green, OH USA; 6 Parker Owens Research Group, Frisco, TX USA

**Keywords:** African american, Young adults, HIV, PrEP, Sociocultural, Structural

## Abstract

**Background:**

Pre-exposure prophylaxis (PrEP) demonstrates effectiveness in decreasing new cases of HIV. However, few African Americans use PrEP, despite being disproportionately impacted by HIV. Understanding the influence of sociocultural and structural factors on PrEP use among multiple priority groups of African Americans, including but not limited to men who have sex with men, may improve PrEP engagement and uptake. The social ecological model (SEM) as a framework guided the understanding of how these factors operate on multiple levels to influence PrEP use among this population.

**Methods:**

This study derived data from the Afya PrEP study consisting of eleven focus groups (N = 63) with 18-29-year-old African American sexual and gender minority and heterosexual individuals at heightened behavioral vulnerability to HIV. We employed constructivist grounded theory processes to inductively analyze the data. A pooled kappa score of 0.90 indicated excellent inter-rater agreement.

**Results:**

Factors impacting PrEP engagement among African American young adults included: (1) Community/social network influences; (2) medical mistrust; (3) stigma; (4) PrEP availability and accessibility, which had two sub-categories: (a) cost and (b) where to obtain PrEP; and (5) PrEP engagement strategies, which had two sub-categories: (a) current AIDS service organizations’ PrEP engagement practices and (b) recommended future PrEP engagement strategies. Categories one through three represent sociocultural factors, and categories four and five represent structural factors that influence perceptions and attitudes of African American young adults regarding PrEP.

**Conclusion:**

Our study highlights sociocultural and structural factors that act as barriers and facilitators to PrEP engagement. The SEM guided the understanding of how these factors operated on multiple levels. One of the sociocultural factors, community/social network influences operated at the interpersonal level of the SEM; the other two, stigma and medical mistrust, operated at the community level. The structural factors (PrEP availability, accessibility, and engagement strategies) operated at the institutional/organizational level. Thus, multi-level interventions are warranted to improve PrEP engagement among various African American young adult priority groups.

## Background

Pre-exposure prophylaxis (PrEP), an HIV prevention drug approved by the FDA [1] is highly effective in decreasing new infections [2]. However, few African Americans, youth, and women use PrEP [3]. Out of the 1.2 million PrEP-eligible individuals, 23% have been prescribed PrEP, and only 8% are African American [4]. Yet, African Americans are disproportionately affected by HIV in the United States (U.S.) as they make up 42% of newly diagnosed cases annually in the U.S [5]. The CDC estimates that 1 in 2 African American men who have sex with men (MSM) compared to 1 in 11 White MSM and 1 in 48 African American women compared to 1 in 132 White women will receive a positive HIV diagnosis in their lifetime [6]. Also, African American transgender women account for 46% of new HIV diagnosis among transgender women [7]. Examining factors contributing to disparities, such as those influencing HIV prevention through PrEP among African Americans, is warranted.

African Americans are likely to entertain negative beliefs and suspicions about PrEP use compared to other racial groups (such as Whites or Latinx), partly explaining low PrEP use among African Americans [8, 9]. While intrapersonal factors are important predictors of behavior, these behaviors are influenced by social and physical environments that may restrain or promote those behaviors [10].

Social determinants of health (SDOH) have been linked to HIV-related outcomes, such as HIV testing, diagnosis, and engagement in HIV care [11–16]. Sociocultural processes, including knowledge of HIV prevention strategies in one’s social network, anticipated HIV-related stigma within one’s community, and medical mistrust, can reduce the likelihood of engaging in HIV testing and linkage to care [14, 17, 18]. In addition, structural factors such as cost, interactions with health care providers, and PrEP availability and accessibility have implications for PrEP engagement and uptake among African Americans [8, 9, 19–21]. Research further demonstrates that sociocultural, socioeconomic, and systemic/structural factors are often far more critical determinants of PrEP use and willingness to use PrEP among African Americans than individual factors like knowledge and awareness [8]. This underscores the need for a multi-level approach to understanding and addressing factors impacting PrEP use among African American groups.

The Social Ecological Model (SEM) provides the basis for observing health outcomes at multiple levels of influence and is based on the premise that physical, social, and environmental influences may play a pivotal role in health and disease outcomes among individuals [22]. The SEM recognizes that individuals are part of and influenced by their larger environments and social systems; also, health outcomes or behaviors of individuals are influenced by several factors existing at multiple dimensions or multiple levels within those environmental contexts [23, 24]. As applied in health promotion, the SEM consists of five levels: individual or intrapersonal (e.g., attitudes and knowledge, behavior control), interpersonal (family, peer groups, sexual networks), institutional or organizational (e.g., cost, provider access, access to health care), community or societal (e.g., cultural beliefs, myths, stigma, homophobia, discrimination, etc.), and public policy level, with the understanding that factors may overlap across these levels [25, 26]. Studies have shown the salience of ecological approaches in understanding barriers and facilitators to PrEP uptake. Philbin et al. [27] assessed factors impacting PrEP use among Black MSM across multiple levels — individual, interpersonal, community, and structural levels. They demonstrated that exploring factors influencing low PrEP uptake at more than one level is important for developing effective interventions to successfully address such factors among vulnerable groups like African American MSM. Researchers call for more investigations emphasizing a better understanding of multi-level factors that influence PrEP access and uptake among African Americans [9, 20, 28]. However, multi-level determinants of PrEP use are not adequately examined among multiple priority groups of African American young adults beyond men who have sex with men (MSM) [19, 27–30]. Only a few PrEP studies have focused on cisgender heterosexual women and men [21, 31–34] and cisgender African American women [35]. Even fewer studies target African American transgender persons [8, 36]. To fill this gap, we assessed the sociocultural and structural contexts that may serve as barriers or facilitators to PrEP engagement and uptake among a diverse sample of African American cisgender heterosexual and LGBTQ + young adults. The SEM served as an appropriate guiding framework to examine multiple factors imbedded within the complex contexts of both the social and structural environments of African American groups. The SEM also provided a better understanding of how these multi-level factors operate, namely at the interpersonal, institutional, and community levels of the SEM. Moreover, prior to the Afya study (a multi-level intervention to improve PrEP awareness and access among African Americans) from which data for this analysis were derived, no study, to our knowledge, examined multi-level factors impacting PrEP use among multiple African American groups of young adults with high HIV vulnerability in Louisville Kentucky. The current analysis expands on previous work that assessed intrapersonal and interpersonal level factors influencing intentions to use PrEP among this population [37].

## Methods

This study derived data from the Afya study that employed a multi-level approach for increasing PrEP awareness, acceptability, and access among African Americans in Louisville, Kentucky. Study methods that highlight both rigor and trustworthiness of the analysis approach have been previously reported [37].

### Sampling, data collection, and data analysis

Eleven focus groups (N = 63) (Table 1) lasting 35–70 minutes long were conducted from August to November 2018 with 18-29-year-old African Americans who self-identified as (i) sexual and gender minorities (LGBTQ+), and (ii) heterosexuals engaging in behaviors that heighten their HIV vulnerability (e.g., having sex without condoms with partners of unknown HIV status, serodiscordant couples, or commercial sex workers). Participants were recruited through various venues, including churches, house balls, pride festivals, community-based organizations (CBOs), and local businesses, and through respondent-driven sampling [38].

**Table 1:** Focus Group Demographics

Group #	Group Type	N
1	Exclusively cisgender MSM	3
2	Combined group, cisgender heterosexual & MSM	9
3	Exclusively trans people & MSM (gender and sexual minority)	4
4	Combined group, heterosexual & MSM & trans	4
5	Exclusively cisgender heterosexual men	3
6	Exclusively cisgender heterosexual women	7
7	Exclusively cisgender heterosexual women	4
8	Combined group, cisgender heterosexual women & men	5
9	Combined group, cisgender heterosexual women & men	7
10	Combined group, cisgender heterosexual women & men	12
11	Combined group, cisgender heterosexual women & men	5
N	Total Number of Participants	63

Participant characteristics were used to determine focus group type (e.g., cisgender heterosexual identifying women were placed in a group with those characteristics). There were some focus groups (Table 1) with similarities in gender identity or sexual orientation to improve group dynamics, and other groups had a combination of characteristics to enhance the diversity of perspectives [39].

A team of experts in HIV and qualitative methods developed and pilot tested a semi-structured focus group guide informed by the SEM. The focus group guide (a portion of which has been previously published) [40] captured a range of specific priority topics and some emerging topics, all of which operated at various levels of the SEM, intrapersonal, interpersonal, institutional, and community levels. Topics included PrEP awareness, knowledge, and attitudes [37], communication for PrEP promotion [40], barriers and facilitators to PrEP, and the role of AIDSs service organizations in PrEP. To elicit various barriers and facilitators to PrEP, participants were asked, *“What would make it easier for people at risk for HIV to get PrEP?”* To explore some of the structural factors impacting PrEP, the role of AIDS services organizations in PrEP service delivery was explained to participants and a list of local organizations was also provided; then participants were asked, *(a) “How can AIDS service organizations (ASOs) break down barriers to getting PrEP? (b) What are some best strategies that ASOs can use for promoting PrEP use among young people?*

Focus groups were conducted by a predominantly African American team trained and experienced in qualitative data collection. We were intentional about hiring facilitators who had worked with young people, sexual minority groups, and African Americans. Each participant was compensated $35, and if they recruited their peers, the participant received $10 extra (up to $30 max) for each peer. The study received approval from the University of Louisville Institutional Review Board. The research team kept memos, took notes during focus group sessions, and debriefed afterwards to improve facilitation of subsequent sessions, all of which contributed to increasing the rigor and trustworthiness of the process [41–43].

We employed a regiorous and systematic process to inductively analyze the data using constructivist grounded theory strategies (initial and focused coding) [43] to generate themes. Initial coding was completed on four out of 11 transcripts that allowed codes to emerge from the original data. These codes were combined and grouped into focused codes that were clearly defined to highlight the underlying properties of each code. Two team members worked independently and together, and through a process of consensus building and peer debriefing negotiated code definitions, refined codes, and finalized the codebook. Some sub-codes were derived to create subcategories within the main focused codes for an in-depth analysis of the data. The final codebook was then applied all transcripts in Dedoose, a qualitative analysis software [44]. Other team members also reviewed data analysis for code application accuracy to increase credibility of the analysis process. Inter-rater agreement was determined using a pooled kappa score of 0.90, indicating excellent agreement [45].

## Results

**Fig. 1:**
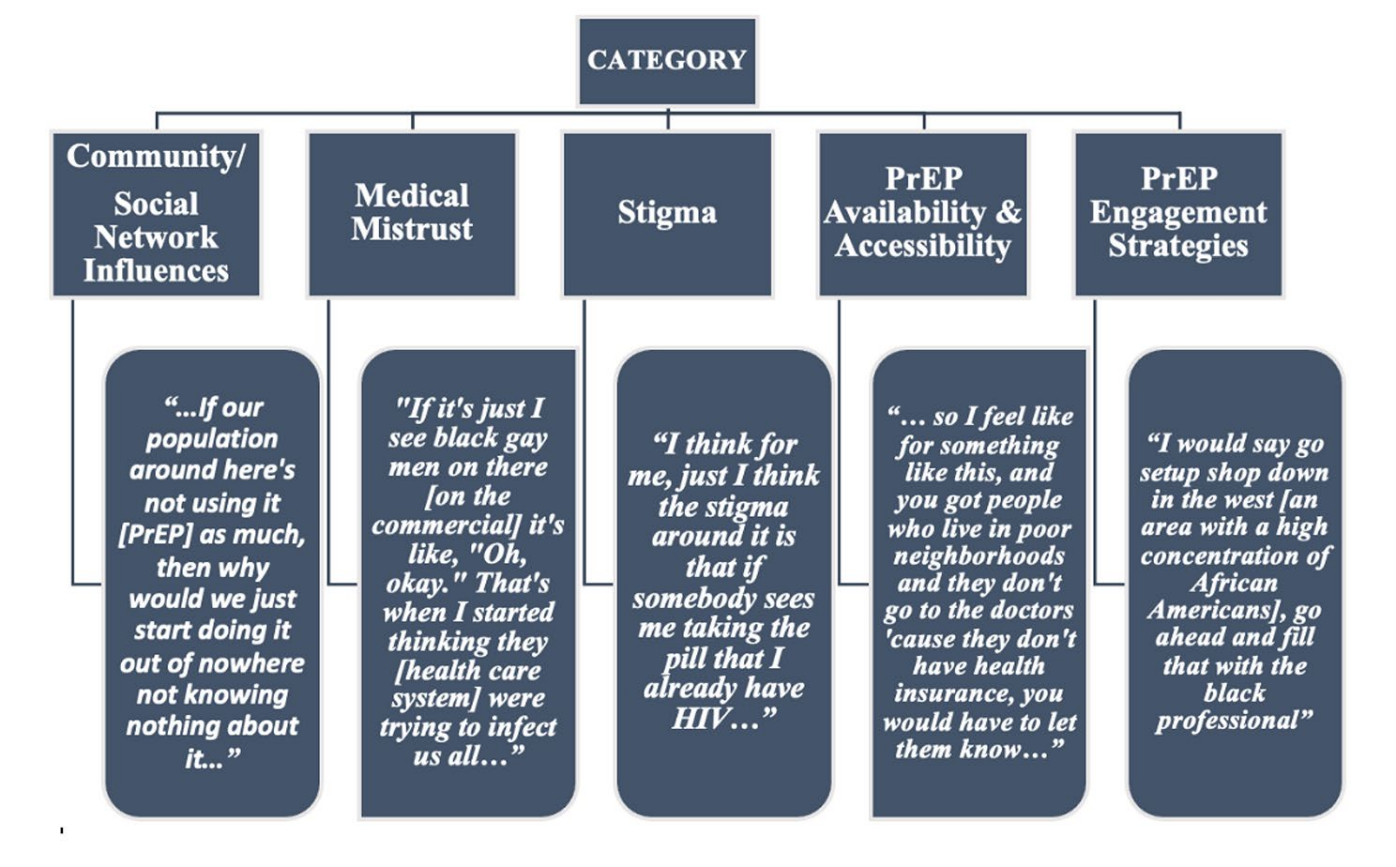
Sociocultural and Structural factors impacting PrEP engagement for Priority Groups

All focus group participants were African Americans, 18–29 years old who resided in Louisville, KY (Table 1). We describe themes (Fig. 1) that emerged from the data analysis in five main categories, three sociocultural: (1) Community/social network influences; (2) medical mistrust; (3) stigma; and two structural: (4) PrEP availability and accessibility, which had two sub-categories: (a) cost and (b) where to obtain PrEP; and (5) PrEP engagement strategies, which had two sub-categories: (a) current ASOs’ PrEP engagement practices and (b) recommended future PrEP engagement strategies. Any across-group differences are broadly highlighted and discussed across themes.

### Community/social network influences

Participants suggested that the larger African American community influences intentions or decision making around PrEP. Some participants indicated that if PrEP was not commonly used within the larger African American community, the participants and other people within their community would be hesitant. One person stated,

I feel like if a lot of African Americans aren’t using it as much, then why would everybody else use that’s African American too? If our population around here’s not using it as much, then why would we just start doing it out of nowhere not knowing nothing about it—Participant, combined group, cisgender heterosexual men & MSM

Consequently, some participants alluded that opinions and behaviors of trusted persons within the participants’ community or social network such as their friends would most likely influence participants’ perceptions of and predispositions toward PrEP compared to others outside their community. One person said,

I just felt like if it was me, there’s my friends and there’s a whole bunch of frat dudes, white fraternity dudes just doing that. And they’re telling me, “Oh do this, and do that.“ Like I’m not gonna believe them. — Participant, combined group, cisgender heterosexual men & MSM

Additionally, participants expressed that if PrEP were endorsed by influential persons who are African American such as well-known public figures or celebrities who are admired by young African Americans, they will likely consider PrEP. They implied that African American young people easily relate to people whom they trust and who are popular within the African American community. One person said,

So, like, Queen Latifah [Black celebrity], you know that’s very important, I think I would really like to see her on something like that [PrEP promotion]. I know we’re thinking of rappers and stuff like that but I’m really just thinking of public figures…like people in my community, you know what I’m saying, who I see every day doing stuff, people who I look to and be like, “Oh they said that, it must be true,” you know what I’m saying I want those people on the posters.— Participant, exclusively cisgender heterosexual women

### Medical mistrust

Medical mistrust, expressed as misgivings about PrEP and suspicion of pharmaceuticals or biomedical interventions, was mainly prevalent among male participants (both heterosexual and MSM). Participants were suspicious of PrEP promotion efforts, particularly how heavily LGBTQ + people were targeted through PrEP television commercials. Some speculated and endorsed conspiracy beliefs alluding that the pharmaceutical industry’s intent on targeting African American gay men was to infect them with HIV. One person noted,

…It’s just targeting me as a black gay man. If it’s just I see black gay men on there [on the commercial] it’s like, “Oh, okay.“ That’s when I started thinking they [pharmaceutical industry] were trying to infect us all. — Participant, combined group, cisgender heterosexual men & MSM

This mistrust of PrEP impacted some participants’ willingness to use PrEP to the extent that some male participants did not trust their doctor’s advice about PrEP. One person stated

Yeah, my doctor just kinda brought it up to me. He was just like, ‘Are you sexually active?’ And I was like, ‘Yeah.’ And instead of him asking my lifetime number, which is what I’m used to kinda hearing, he was just like, ‘Are you on PrEP?’ And I was like, ‘No, and I don’t know about getting on it.’ Cause I was still kinda leery about it. So, he’s kinda got the ball rolling on it. I still have some reservations. Just about, I don’t trust a lot... — Participant, combined group, cisgender heterosexual men & MSM.

### Stigma

Participants discussed stigma related to HIV, PrEP, and being a person who identifies as gay. Stigma was expressed as anticipated stigma (that is participants’ expectations of society’s judgmental perceptions and attitudes toward persons with HIV, those who use medications associated with HIV, in this case PrEP, as well as persons who identify as gay). Several participants anticipated that people would think badly of a person taking medication to prevent HIV, and that person would potentially be targeted, judged, or misconstrued as being HIV positive. One person said,

I think for me, just I think the stigma around it is that if somebody sees me taking the pill, that I already have HIV and it’s not to prevent it, but it’s that I’m on retroviral treatment. — Participant, combined group, cisgender heterosexual men & MSM

Thus, the anticipation of being stigmatized by others within the community if known to be taking PrEP impacted participants’ attitudes toward PrEP use.

Stigma was also expressed as enacted stigma (acts or behaviors of discrimination against someone perceived to have a stigmatizing condition). Some participants expressed their sentiments or suspicion of persons taking PrEP, suggesting that taking the medication may prompt mistrust or judgement from others within the community or social networks. One person noted,

If someone was taking it, I’d probably be just thinking, “Why are they taking it? What [are] they doing that they need to take the pill? So that would be just my question.” —Participant, combined group, cisgender heterosexual men & MSM

Further, participants expressed that the excessive targeting of PrEP promotion toward gay individuals perpetuates stigma by leading the public to associate PrEP with persons who are gay. Participants noted that this practice not only shames and stigmatizes gay individuals but also excludes others (particularly men) who may need PrEP but do not identify as gay. One person said,

I think the whole label aspect of it all shames. Sometimes it demasculates some guys if they’re put in a category, the same category with people like myself or like my friend…They don’t want to be labeled as gay men, but they still need that [PrEP] for the same protection, and they need to know that it’s not just subject to the gay community. — Participant, exclusively trans people & MSM (gender and sexual minority)

Overall, the anticipation of being stigmatized for using PrEP appeared to influence participants’ perceptions about accepting the intervention.

### PrEP availability and accessibility

#### a) cost

Participants showed interest in PrEP but were worried about the cost. Many were unaware that most insurance plans covered PrEP and that medication assistance programs are available to reduce the cost burden associated with using PrEP for eligible persons who do not have insurance. One participant stated,

I guess PrEP is good and everything, don’t get me wrong, and I’ve actually even tried it, but I feel like… when I looked up information about PrEP afterwards, with PrEP there was something like it costs $4,000 to get and like you said some insurance cover it, some insurances don’t. I wasn’t sure about which was which and it made me think ... make it seem like it’s easy to get but it isn’t. — Participant, exclusively trans people & MSM (gender and sexual minority)

Similarly, participants reasoned that cost would be a barrier to obtaining PrEP for the poor and uninsured, particularly those who live in neighborhoods that may be out of reach of health promotion activities. Thus, they suggested having outreach and being explicit about how to easily access PrEP at no cost to accommodate the poor and uninsured. One person said,

I feel like for something like this, and you got people who live in poor neighborhoods, and they don’t go to the doctors ‘cause they don’t have health insurance, you would have to let them know. You have to come to them and reach out in their communities to let them know, ‘Look, this is what we’re offering, it’s free, come see us and we’ll give it to you.’ ‘Cause they’re not going to go find out about it. — Participant, exclusively cisgender heterosexual women

#### b) Where to obtain PrEP

Participants saw providers’ offices as facilitators of PrEP engagement, although others (particularly MSM) perceived this as a potential barrier. Cisgender heterosexual women expected to have heard about PrEP during routine health care visits. However, these women were surprised and upset that their PCP did not mention PrEP during regular visits. As one cisgender woman noted, “none of our physicians, none of our primary care providers or doctors or nurses or anybody we talk to are mentioning this to us.” Several women reported presenting for sexually transmitted infection (STI) testing on several occasions and should have heard about PrEP but noted that they were never told about it.

I would say, I usually get my full STD testing from my nurse practitioner when I go to see her every year, and I have also gotten it from the city, and in neither of those experiences have they mentioned PrEP, which is interesting and maybe they don’t ... think I’m at high-risk per se... — Participant, exclusively cisgender heterosexual women

These women implied that they trusted their PCP’s advice and would even feel comfortable talking to their providers about their health. One woman said,

My health care provider, I will say I’m comfortable with her. I’ve asked her questions about stuff that I didn’t want to, but I know I needed to ask, and she didn’t make me feel uncomfortable at all, she just answered my question and give me good advice. This is someone who [inaudible 00:35:42] give you a straightforward answer and not look at you like they’re judging you. — Participant, exclusively cisgender heterosexual women

In sharp contrast to heterosexual women, some men, especially in our MSM group, did not feel comfortable learning about PrEP from a PCP unprompted. They perceived that the health care system’s practice of overly targeting PrEP promotion toward gay individuals perpetuates the stigma and stereotyping of individuals who are gay. Consequently, these participants reacted negatively toward being singled out by health care personnel for PrEP awareness simply because they identify as gay. One participant was infuriated when they were handed a PrEP promotional material (pamphlet) at a provider’s office during a routine visit. They vented,

Stuff like that [targeted PrEP promotion], that’s the people [the heterosexual individuals] who need it. We know about it [PrEP]. Because we’re gay, they’re gonna throw it in our face, we going to the doctor and the nurse gonna slip us a little pamphlet because she think we gay, bitch! —Participant, combined group, heterosexual & MSM & trans people

Participants generally desired to have PrEP readily available at PCP offices and STI/HIV testing clinics. Moreover, they strongly preferred to have all services integrated to normalize HIV, rather than keeping HIV separate, which participants thought perpetuates HIV stigma. One person stated,

HIV is the only thing that’s separate from almost everything else. If you have herpes, gonorrhea, all that, you still go to the same place, but if you have HIV, you are over here. If maybe, they could … move it on over to the rest of the STDs. — Participant, combined group, heterosexual men & MSM & trans people

### PrEP engagement strategies

To inform the improvement of local AIDS service organizations’ (ASOs) strategies for engaging African American priority groups for PrEP delivery/outreach and other HIV prevention services, participants reported on current ASO service delivery practices and recommended future PrEP engagement strategies.

#### a) Current ASOs’ PrEP engagement practices

Participants expressed dissatisfaction with current ASOs outreach efforts. They indicated that ASOs’ presence was not felt within their community. Many participants were unaware of these organizations’ existence and actions around PrEP within the community. Specifically, participants were frustrated that many resources were out of reach of young African Americans and located mostly downtown or in predominantly White neighborhoods (such as on the east end of Louisville) that require long commute times. They reported that barley any sexual health resources were located on the west end of the city (predominantly occupied by African Americans). One person noted,

Not even just downtown, but there’s also places…towards the east end…that have all these resources and yet these resources are only stuck in one area that’s very difficult to get to, especially by bus that take practically an hour or two if you miss it... If there was more places where they could be actually reachable to younger people then it would be a hell of a lot more easier to even take care of themselves… — Participant, exclusively trans people & MSM (gender and sexual minority)

Additionally, participants reported that current (at the time of the study) promotional efforts did not resonate well with them. They thought current commercials were non-representative of African Americans and overly focused on sexual transmission of HIV, but not other modes of transmission.

I think if they advertise it, not just through sex as well, because that commercial is like, it’s very, sex oriented and you can get HIV in various different forms, ways, whatever. Oh, it was White too…, just the commercial alone, that I’ve seen on YouTube, it was White. — Participant, exclusively cisgender heterosexual women

#### b) Recommended future PrEP engagement strategies

Participants indicated they would be more likely to accept PrEP-related information if it was recommended by a trusted person, preferably someone who looks like them (African American), or at least someone with whom they could relate. Participants strongly advised against employing personnel of a different race to deliver PrEP messages to African Americans. Some participants did not feel like they could identify with someone who was not African American because the participants did not believe that non-African American personnel, especially if they were White, could understand the struggles of an African American person.

I have to be honest with them. Please don’t give me a White person. Please don’t, ‘cause they could never understand my struggle and what the hell I’m going through. I cannot talk to this White person. — Participant, exclusively cisgender MSM

Despite their recommendation to hire PrEP outreach personnel from the African American community, participants cautioned against hiring someone simply because they fit the recommended demographic. Participants expected the hired African American personnel to be qualified for the position — knowledgeable and professional.

For me I would rather see a White professional not pay me attention and do their job than a black person sit in front of me and not be professional... — Participant, exclusively trans people & MSM (gender and sexual minority)

Finally, participants suggested that ASOs should consider situating their offices in places that are easily accessible to young African Americans, make PrEP services available at neighborhood clinics, and incorporate services into routine care such as STI services.

I would say go setup shop down in the west [an area with a high concentration of African Americans], go ahead and fill that with the black professional… — Participant, exclusively trans people & MSM (gender and sexual minority)

Overall, participants wanted to see more ASO engagement within the African American community. They believed ASOs could improve their PrEP service delivery and engagement with young African Americans by establishing their presence within reach of the community, building rapport with, and hiring personnel who identify with and relate to young African Americans’ struggles.

## Discussion

This study highlights sociocultural and structural factors identified by African American young adults that influence perceptions and attitudes toward PrEP use among them. Themes inductively derived from analysis of focus group data were based on commonalities of issues raised by all participant groups. Understanding nuances behind reticence to PrEP engagement among young African Americans is a necessary step toward reducing PrEP use disparities.

The social ecological model (SEM), as a guiding framework, provides the basis for understanding and contextualizing our findings. The SEM demonstrates how factors impacting health outcomes operate at multiple levels. In the case of this study, findings appeared to operate at three levels of the SEM: interpersonal, organizational (institutional), and community levels. Community/social network influences operated at the interpersonal level, others (stigma, medical mistrust) at the community level, and the structural factors (PrEP availability, accessibility, and engagement strategies) operated at organizational level of the SEM to influence perceptions and attitudes toward PrEP use among African American young adult priority groups (heterosexual men and women, in addition to LGBTQ+) in Louisville, KY.

### Interpersonal level

At this level of the SEM, “Interpersonal relationships with family members, friends, neighbors, contacts at work, and acquaintances are important sources of influence in the health-related behaviors of individuals” [26]. Our findings at this level revealed how participants’ interactions within the larger African American community and with their social or sexual networks influenced PrEP engagement. For instance, some participants believed they would be more likely to use PrEP if it was widely accepted and endorsed as an HIV prevention option by the larger African American community as well as by African American influencers and popular public figures. Additionally, some participants implied they would be more likely to believe trusted persons within their community or social networks who approve of PrEP and recommend it to them. This finding reinforces evidence suggesting that injunctive norms (perceptions of who approves or disapproves of a behavior) have an impact on PrEP use [46, 47]. This has implications for PrEP promotion efforts to target trusted community or social network members of African American young adults who may influence decision making among them. Since young people are likely to trust the opinions of their peers [48], if trusted individuals within the community and social networks of these young adults endorse PrEP, chances are they will follow suit.

### Organizational (institutional) level

The organizational level of the SEM includes factors like formal or informal rules, regulations, policies and practices within an organization which may influence behavior change. These characteristics are important as institutions or organizations can play a vital role in health outcomes of individuals. Organizations may serve as targets for spearheading activities and diffusing health promotion programs [26]. In our study, organizational level factors included PrEP availability and access as well as provider preferences and expectations which appeared to shape PrEP engagement and willingness to use PrEP. Cisgender heterosexual women in our study expected their primary health care providers to have informed them about PrEP. They expressed frustration that their PCPs never mentioned nor recommended PrEP during routine health visits. The failure of health care providers to adequately provide PrEP information to or address PrEP needs of African Americans, especially women, has been previously documented [20, 49] and is worrisome. The fact that women in the study expect their PCP to tell them about PrEP indicates that these women would likely trust PrEP information from their provider. This finding has positive implications for PrEP implementation, especially in primary care settings frequented by African American women. In previous studies, African American women trusted their primary care providers to provide PrEP services and were willing to use PrEP if a provider recommended it [21, 32]. However, our findings and evidence from previous research revealed that women and their providers may have limited PrEP knowledge [50]. This finding underscores the need to equip providers catering to women, such as those in reproductive health care settings, to orient and educate their female clients about HIV vulnerability and PrEP. Increasing PrEP awareness among women holds the potential for increasing their PrEP uptake and thus decreasing HIV vulnerability [51], especially since African American women are the group with the second highest vulnerability to HIV after MSM and bisexual men of all races [52].

In contrast to cisgender women, MSM and some heterosexual men in our study were not positively inclined toward receiving PrEP education from their PCP unprompted. Efforts by health care providers to promote PrEP out of the blue, such as being handed a flyer about PrEP during an unrelated office visit, was perceived as stigmatizing and stereotyping to MSM. This finding has implications for PrEP engagement and access among African American men, especially MSM. Public health messages that destigmatize and normalize the potential benefits of PrEP for all individuals, irrespective of sexual and gender identity, who use condoms inconsistently and have concurrent sexual partners and/or those with unknown HIV status [53] may help improve attitudes toward PrEP among cisgender heterosexual and LGBTQ + African American young men engaging in high-risk behaviors. Other studies have documented that African American men are uncomfortable discussing or disclosing their sexual behaviors with their providers [9, 54], stemming from medical mistrust and perceived risk of discrimination [54]. This calls for more efforts targeted toward understanding and addressing African American young men’s preferences for accessing PrEP services. Determinants of access within the environments of the target population, particularly specific priority groups, would need to be taken into consideration if PrEP intervention is to be effective; otherwise, the efforts are pointless [55].

Further, our findings highlighted reactions toward current PrEP engagement strategies of AIDS service organizations (ASO) and recommendations for future efforts. Participants were unsatisfied with current outreach strategies for engaging young African Americans with PrEP. Many did not feel ASOs had a strong enough presence within the community. They also did not perceive that PrEP outreach efforts resonated well with African American priority groups. They wanted HIV prevention and PrEP promotional messages to be more inclusive of various risk categories and tailored more to African Americans. Additionally, participants recommended PrEP integration into existing sexual health services offered at accessible locations within their neighborhoods for ease of access. Moreover, participants strongly desired to see more African Americans or peers of priority groups employed by ASOs to conduct HIV/PrEP outreach within the African American community. Evidence suggests that peer-to-peer education is effective for HIV prevention initiatives [48]. Participants believed this practice would make ASOs more relatable and trustworthy and thus improve engagement with PrEP services and PrEP uptake among young African Americans.

Additionally, structural factors such as cost were discussed by participants and shown to influence their decision making. Participants demonstrated interest in PrEP but were concerned about affordability. However, concerns about cost were born from a lack of awareness that PrEP is covered by insurance and that medication assistance programs also exist to defray costs associated with initiating PrEP by uninsured participants. This underscores the need for awareness campaigns and outreach efforts by ASOs geared toward African Americans to highlight information about PrEP access, such as cost information and coverage by most insurance formularies.

### Community level

The community level of the SEM involves social, cultural, and societal norms and may leverage relationships among organizations and institutions for influencing behavior [56]. In our study, significant findings at this level included medical mistrust and stigma. Medical mistrust was more apparent among participants who identified as men. Several of them did not trust PrEP and some insinuated that the pharmaceutical industry might be targeting African American men to infect them with HIV. This caused some men to be suspicious of using PrEP. Other studies also demonstrated that deeply expressed concerns about stigma, endorsement of conspiracy beliefs, and medical mistrust considerably impacted PrEP-use intentions among African American MSM [8, 9, 19, 30, 57]. It is plausible that this prevalence of mistrust among these participants is due to the lingering effects of historical unethical research improprieties like the Tuskegee Syphilis study, that mainly focused on African American men [58]. Evidence further reveals that African American men experience unfair treatment and discrimination in society, and particularly within the health care system [59, 60]. In one study, many participants reported instances when they or people they knew were treated disrespectfully or poorly (e.g., not being given the proper medical tests) because they were Black [59]. The health care industry will need to consciously assess and address factors that perpetuate distrust among African Americans to improve health care access, especially among men.

Another finding in the study that operated at the community level was stigma which played a key role in how participants perceived PrEP. Participants reported that people within their community would associate PrEP with HIV and feared being misconstrued as having HIV if they were seen using PrEP. Additionally, PrEP was erroneously associated with MSM partly due to the excessive targeting of PrEP advertisements toward LGBTQ + individuals. As a result, some heterosexual individuals in the study did not want to be seen taking PrEP, nor did they perceive themselves as being vulnerable to HIV or in need of PrEP. This behavior is in concert with evidence suggesting that if individuals do not associate with characteristics of the persons to whom the behavior is linked, they will not engage in the behavior [46]. This has implications for persons who do not identify with labels, particularly African American men who have sex with other men but who may not identify as gay or MSM and whose sexual behaviors may place them at elevated HIV vulnerability. Prior research has shown that non MSM-identifying young African American men who have sex with men but who identified as heterosexuals have been left out of HIV prevention activities because they did not identify as gay [54]. Therefore, addressing the unintended consequences of associating PrEP with LGBTQ + individuals in future PrEP publicity is vital to reduce stigma that has been shown to impact vulnerability to HIV and PrEP uptake among African Americans [57]. Interventionists should critically consider these findings by designing more inclusive PrEP publicity that represents all demographics of persons demonstrating vulnerability to HIV.

### Limitations

Study limitations are noted. First, our findings are specific to our study setting and might not be readily applicable to different contexts. However, the study may have common themes that resonate with African American young adults in other contexts. Second, participants’ social desirability bias likely impacted how participants responded to focus group discussion prompts despite the encouragement of individual contributions. Lastly, not all priority groups were evenly represented (particularly MSM and trans individuals who are considered hard-to-reach) hence some groups were small (e.g., n = 3). We recognize the limitation in the representation in numbers of some of our groups, and we worked with a lot of people to recruit, including leaders in the queer community. We also hired an African American, same gender loving man to help us reach this population, but we did not get the full representation in numbers that we hoped which impacted the composition (limited homogeneity) of some of our groups. Thus, we encourage future studies targeting specific groups of African Americans to assess factors that may be unique to these groups that can be beneficial for tailoring PrEP messaging to them.

## Conclusion

Our study highlights sociocultural (community/social network influences, stigma, medical mistrust) and structural factors (PrEP availability, accessibility, and PrEP engagement strategies) operating at multiple levels of the social ecological model that act as both barriers and facilitators to PrEP engagement. These multi-level factors should be the focus for tailoring outreach to improve PrEP engagement with various African American young adult priority groups. Ecological approaches, which situate behavior within the contexts of the social and physical environments, are more efficacious for effecting long-term behavior change such as is needed in STI/HIV prevention science [10, 56]. Thus, to address multi-level factors such as interpersonal and community (societal) level factors impacting PrEP uptake among African Americans, future research should consider working directly with African American communities (leveraging social networks) to co-develop strategies for decreasing stigma, medical mistrust, and conspiracy beliefs among young African Americans. Additionally, to intervene at the organizational (institutional) level, PrEP should be integrated into routine primary care practice (especially those who serve cisgender women) to improve awareness and access, which can boost uptake. AIDS services organizations should hire more African American individuals from the local communities who can draw upon insider community knowledge to assist ASOs to establish their presence within the community and scale up PrEP outreach efforts.

Furthermore, PrEP promotion interventions should be developed in ways that do not stigmatize any segment of the population but rather strive to normalize PrEP as well as increase perceptions of HIV vulnerability and PrEP need among African American priority groups.

## Data Availability

The datasets generated and/or analyzed during the current study are not publicly available due to the stigmatized nature of the disease, the limited scope of geographic area, the specifics of the content of statements within the data which can lead to participants being more easily identifiable in the local area, data are not publicly available, but are available from the collaborating author, JK on reasonable request.

## Abbreviations

PrEP: Pre-exposure prophylaxis
HIV: Human Immunodeficiency virus
FDA: Federal drug administration
ASO: AIDS service organizations
SDOH: Social determinants of health
LGBTQ+: Lesbian Gay Bisexual Transgender Queer +
CBOs: Community-based organizations
